# CXCL10 Acts as a Bifunctional Antimicrobial Molecule against *Bacillus anthracis*

**DOI:** 10.1128/mBio.00334-16

**Published:** 2016-05-10

**Authors:** Katie R. Margulieux, Jay W. Fox, Robert K. Nakamoto, Molly A. Hughes

**Affiliations:** a Division of Infectious Diseases, Department of Medicine, University of Virginia, Charlottesville, Virginia, USA; b Department of Microbiology, Immunology, and Cancer Biology, University of Virginia, Charlottesville, Virginia, USA; c Department of Molecular Physiology and Biological Physics, University of Virginia, Charlottesville, Virginia, USA

## Abstract

*Bacillus anthracis* is killed by the interferon-inducible, ELR(−) CXC chemokine CXCL10. Previous studies showed that disruption of the gene encoding FtsX, a conserved membrane component of the ATP-binding cassette transporter-like complex FtsE/X, resulted in resistance to CXCL10. FtsX exhibits some sequence similarity to the mammalian CXCL10 receptor, CXCR3, suggesting that the CXCL10 N-terminal region that interacts with CXCR3 may also interact with FtsX. A C-terminal truncated CXCL10 was tested to determine if the FtsX-dependent antimicrobial activity is associated with the CXCR3-interacting N terminus. The truncated CXCL10 exhibited antimicrobial activity against the *B. anthracis* parent strain but not the Δ*ftsX* mutant, which supports a key role for the CXCL10 N terminus. Mutations in FtsE, the conserved ATP-binding protein of the FtsE/X complex, resulted in resistance to both CXCL10 and truncated CXCL10, indicating that both FtsX and FtsE are important. Higher concentrations of CXCL10 overcame the resistance of the Δ*ftsX* mutant to CXCL10, suggesting an FtsX-independent killing mechanism, likely involving its C-terminal α-helix, which resembles a cationic antimicrobial peptide. Membrane depolarization studies revealed that CXCL10 disrupted membranes of the *B. anthracis* parent strain and the Δ*ftsX* mutant, but only the parent strain underwent depolarization with truncated CXCL10. These findings suggest that CXCL10 is a bifunctional molecule that kills *B. anthracis* by two mechanisms. FtsE/X-dependent killing is mediated through an N-terminal portion of CXCL10 and is not reliant upon the C-terminal α-helix. The FtsE/X-independent mechanism involves membrane depolarization by CXCL10, likely because of its α-helix. These findings present a new paradigm for understanding mechanisms by which CXCL10 and related chemokines kill bacteria.

## IMPORTANCE

Chemokines are a class of molecules known for their chemoattractant properties but more recently have been shown to possess antimicrobial activity against a wide range of Gram-positive and Gram-negative bacterial pathogens. The mechanism(s) by which these chemokines kill bacteria is not well understood, but it is generally thought to be due to the conserved amphipathic C-terminal α-helix that resembles cationic antimicrobial peptides in charge and secondary structure. Our present study indicates that the interferon-inducible, ELR(−) chemokine CXCL10 kills the Gram-positive pathogen *Bacillus anthracis* through multiple molecular mechanisms. One mechanism is mediated by interaction of CXCL10 with the bacterial FtsE/X complex and does not require the presence of the CXCL10 C-terminal α-helix. The second mechanism is FtsE/X receptor independent and kills through membrane disruption due to the C-terminal α-helix. This study represents a new paradigm for understanding how chemokines exert an antimicrobial effect that may prove applicable to other bacterial species.

## INTRODUCTION

Infections caused by Gram-positive and Gram-negative bacteria are a major cause of morbidity and mortality worldwide (1). A wide variety of strategies have evolved to prevent or treat these infections. With the global increase in multidrug-resistant bacterial pathogens, current antibiotic therapies are becoming less reliable, highlighting the need for the development of new treatment strategies. Among a wide variety of molecules being studied as novel antimicrobials, chemokines represent a distinct class of proteins. Chemokines are small proteins, 8 to 10 kDa, known best for their highly potent chemoattractant properties that play an immunomodulatory role within both the innate and adaptive immune systems (2–6). Chemokines were initially studied for their ability to attract adaptive immune cells to sites of injury or inflammation. However, they also play a key role in a variety of physiological processes, such as angiogenesis and hematopoiesis (7–12). Interestingly, over the past 20 years, there has been increasing recognition of a direct antimicrobial effect of multiple chemokines (13–19). While antimicrobial activity of a variety of chemokines against Gram-negative and Gram-positive bacteria, viruses, and fungi has been reported, the mechanism(s) through which that effect is achieved remains widely uncharacterized.

CXCL10 is one of the interferon-inducible, Glu-Leu-Arg-negative [ELR(−)] CXC chemokines, a group that also includes CXCL9 and CXCL11 (20). It exerts direct antimicrobial activity against a wide variety of Gram-negative and Gram-positive bacteria such as *Escherichia coli*, *Staphylococcus aureus*, *Listeria monocytogenes*, and *Bacillus anthracis* (14–17). Additionally, CXCL10 exerts an antimicrobial effect against both laboratory strains and clinical isolates of *E. coli*, including a multidrug-resistant strain obtained from a clinical sample (21). Like the structure of other chemokines, that of CXCL10 consists of a relatively unstructured N terminus, three antiparallel β-sheets, and a C-terminal amphipathic α-helix (22–24). The interferon-inducible, ELR(−) CXC chemokines interact with CXCR3, a receptor that is found on host immune cells such as T-cells, B-cells and natural killer cells (25, 26). These chemokines bind to the receptor via a series of interactions that are dependent upon the interaction of the chemokine N terminus with the extracellular domain of CXCR3 (amino acids [aa] 1 to 35) (8, 27). Further interaction of the chemokine N terminus and β-sheets with other regions of CXCR3 then occurs in order to activate the signaling cascade necessary to initiate chemotaxis of host immune cells (8, 26, 27).

*B. anthracis* is a Gram-positive, spore-forming bacterium that causes the disease anthrax following the exposure of susceptible mammals to spores and their subsequent germination into vegetative bacilli (28–31). Our laboratory has previously shown that CXCL10 exhibits *in vitro* antimicrobial activity against both *B. anthracis* spores and vegetative cells (17, 32). *In vivo* neutralization of CXCL10 resulted in a significantly higher mortality rate in a mouse lung infection model with C57BL/6 mice, which are inherently resistant to intranasal infection by the *B. anthracis* Sterne strain (32). Blocking of CXCR3 resulted in no difference in survival from control (vehicle-treated) mice (32). These *in vivo* data support a direct antimicrobial role for CXCL10 that is unrelated to its interaction with CXCR3 and suggest that CXCL10 is produced locally at a high enough concentration to actively kill the bacteria at sites of infection. Given the charge and structural similarity of the CXCL10 C-terminal α-helix to cationic antimicrobial peptides, it was initially hypothesized that the α-helix was responsible for its antimicrobial activity, likely via membrane disruption based on characterized mechanisms utilized by cationic antimicrobial peptides like LL37 (19, 33, 34).

Our laboratory previously screened a *B. anthracis* Sterne strain transposon mutant library to identify bacterial gene products associated with the antimicrobial activity of recombinant human CXCL10 (17, 32, 35). Of particular interest was the finding that disruption of the *ftsX* gene, which encodes FtsX, resulted in the resistance of *B. anthracis* vegetative cells to CXCL10 (35). FtsX is a conserved membrane component of the FtsE/X complex that appears to be a prokaryotic ATP-binding cassette transporter (35–38). The FtsE/X complex in other bacterial species, such as *Bacillus subtilis*, *E. coli*, and *Mycobacterium tuberculosis*, has been shown to play a key role in cell wall peptidoglycan (PG) processing through the activation of PG hydrolytic enzymes by FtsX (37–41). FtsE possesses conserved Walker motifs that are responsible for ATP binding and hydrolysis (40, 42, 43). Work conducted by others using *B. subtilis* and *M. tuberculosis* suggests that ATP hydrolysis by FtsE induces a conformational change in FtsX resulting in the activation of PG hydrolases for cell wall processing, a critical function for maintenance of bacterial cell integrity (39–41). Major classes of antimicrobials (e.g., beta-lactam antibiotics) kill bacteria by inhibiting specific steps in PG synthesis, which also affects bacterial cell integrity (44).

*In silico* analysis of the *B. anthracis* FtsX protein revealed that a 27-aa region (aa 54 to 80) in the large external loop has 45% similarity to the N-terminal amino acid sequence of CXCR3 (aa 9 to 35), the mammalian receptor for CXCL10 (35). Importantly, this N-terminal amino acid sequence is involved in the binding of the N-terminal region of CXCL10 (8, 45, 46). The similarity between CXCR3 and FtsX suggested that the N-terminal region of CXCL10 may also interact with the CXCR3-similar region of FtsX. Thus, the antimicrobial activity of CXCL10 may be primarily the result of a receptor-mediated effect that leads to cell lysis and not necessarily to depolarization resulting from the insertion of the C-terminal α-helix into the cytoplasmic membrane. In the present study, we investigated two potential ways in which CXCL10 may kill bacteria. Our data suggest that the antimicrobial activity of CXCL10 is mediated by its interaction with FtsE/X, which utilizes a mechanism separate from that of the C-terminal α-helix. In the absence of functional FtsE/X, higher concentrations of CXCL10 exhibit a bactericidal effect that appears to be dependent on the presence of its C-terminal α-helix and general membrane depolarization. However, the result of each pathway (i.e., cell lysis) is the same, with killing of *B. anthracis* being the most effective when both mechanisms are present and active.

## RESULTS

### The CXCR3-similar region of FtsX is involved in CXCL10 interaction with *B. anthracis.*

FtsE/X is widely conserved among Gram-positive and Gram-negative bacterial species, with a similarity ranging between 46 and 75%, as determined by BLASTP analysis and amino acid sequence alignment (see Text S1 in the supplemental material) (47, 48). In general, bacterial FtsX protein structures consist of four transmembrane domains and two extracellular loops, although functional differences between the FtsX proteins of Gram-positive and Gram-negative bacterial species have been observed (35, 42). In Gram-positive bacteria, FtsX, along with FtsE, exists in an FtsE/X complex that is involved in PG processing during cellular elongation and spore formation. In Gram-negative bacteria, FtsX and the FtsE/X complex have been shown to direct PG processing at the bacterial septum during cell division (37–41, 49). Our initial investigation into *B. anthracis* FtsX identified two regions of similarity between FtsX and the known mammalian CXCL10 receptor, CXCR3 (50) (see Text S1). While those specific regions are not conserved between Gram-positive and Gram-negative species, there are other regions of similarity between CXCR3 and FtsX, notably located in the exposed outer loops. In this study, the focus of investigation involved the region of *B. anthracis* FtsX (aa 54 to 80) with sequence similarity to the region of human CXCR3 (aa 9 to 35) that is primarily responsible for chemokine binding, since that similarity between FtsX and CXCR3 suggested a region for possible interaction with CXCL10.

**FIG 1:**
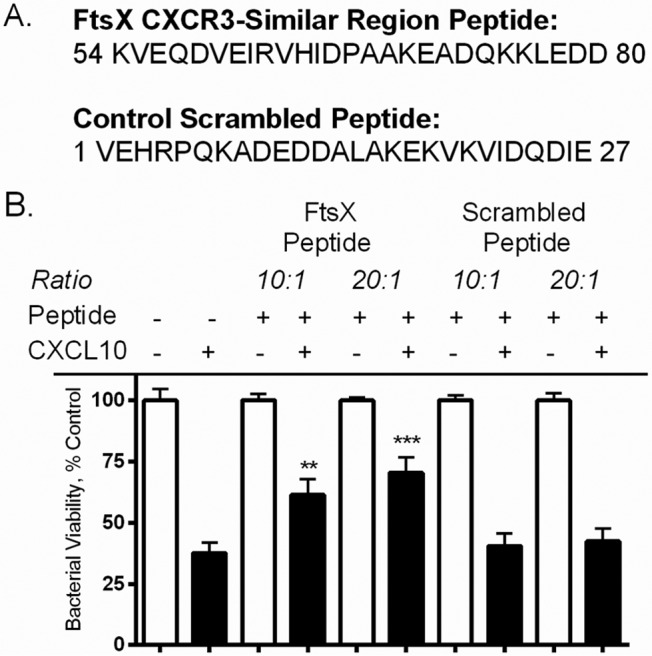
The CXCR3-similar region of FtsX is important in mediating CXCL10 antimicrobial activity against the *B. anthracis* parent strain. (A) A synthetic peptide consisting of the 27 aa (aa 54 to 80) of FtsX with similarity to the CXCR3-binding region (aa 9 to 35) of CXCL10 (35) and a scrambled control. (B) A peptide competition assay with the FtsX peptide in a 10:1 or 20:1 molar ratio with 0.46 µM CXCL10 resulted in statistically significant less killing of the *B. anthracis* parent strain than by CXCL10 alone. Incubation with the control peptide had no effect on killing. Bacterial viability was measured by alamarBlue reduction, and fluorescence is expressed as a percentage of that of the strain-specific untreated control. Data points represent mean values ± the standard errors of the means; *n* = 3 separate experiments using triplicate wells. **, *P* ≤ 0.001; ***, *P* ≤ 0.0001

A peptide competition assay was performed to determine if the CXCR3-similar region of FtsX is involved in facilitating the antimicrobial activity of CXCL10. A 27-aa peptide representing the CXCR3-like region of FtsX was synthesized, as well as a scrambled peptide to control for sequence specificity (Fig. 1A). The antimicrobial activity of CXCL10 alone or the peptide-CXCL10 mixture against the *B. anthracis* Sterne strain, hereby designated as parent strain, was examined after 2 h of incubation (35). There was a statistically significant increase in the survival of bacteria exposed to the FtsX peptide-CXCL10 mixture at molar ratios of both 10:1 and 20:1 (Fig. 1B). No significant increase in survival was observed in the scrambled peptide-CXCL10 competition assay (Fig. 1B). These results suggested that the CXCR3-similar region of FtsX was important in mediating the antimicrobial effect of CXCL10 and that the interaction required a specific sequence and was not just due to peptide charge interactions.

### C-terminally truncated CXCL10 (CTTC) synthesis and physical characteristics.

The importance of the CXCR3-similar region of FtsX indicated a possible site for the interaction of CXCL10 with *B. anthracis*. It was previously determined through the work of others that the relatively unstructured N-terminal region of CXCL10 initially interacts with CXCR3, resulting in immune cell activation (8, 27). We hypothesized that the N-terminal region of CXCL10 may also interact with bacterial FtsX, resulting in an antimicrobial effect. The amphipathic α-helix of various chemokines has been considered to be the portion of the proteins responsible for antimicrobial activity because of its positive charge and secondary structural resemblance to cationic antimicrobial peptides and defensins (19, 33, 34). However, since our findings unexpectedly implicated the N-terminal portion of CXCL10 in eliciting an antimicrobial effect, we conducted the following series of experiments.

**FIG 2:**
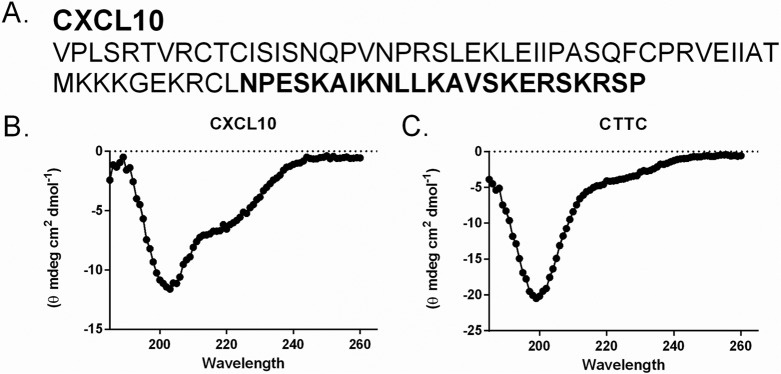
Complete amino acid sequence and secondary-structure comparison of CXCL10 and CTTC. (A) Amino acid sequences of intact CXCL10 and synthetic protein CTTC (aa 1 to 54) lacking the C-terminal α-helix. The amino acids removed from intact CXCL10 are in bold. (B, C) CD showed a distinct α-helical pattern in the intact CXCL10 spectrum, as indicated by peaks at wavelengths of 190 and 210 nm; these peaks were absent from the CTTC spectrum. The calculated β-sheet contents of both the CXCL10 and CTTC spectra were consistent with the estimated percentage of β-sheets in the published CXCL10 crystal structure (45).

To determine if it is the N-terminal region or the C-terminal α-helix of CXCL10 that is responsible for antimicrobial activity against *B. anthracis*, a truncated CXCL10 protein was synthesized that lacked the C-terminal 23 aa (Fig. 2A). The sequence of CTTC was confirmed by mass spectrometry (data not shown). Circular dichroism (CD) spectra confirmed that CTTC lacked the α-helical content, as evidenced by the absence of the spectral peaks at 190 and 210 nm characteristic of intact CXCL10 (Fig. 2B and C). Importantly, analysis of the CTTC spectrum revealed that the content of β-strands (39%) was similar to that of the properly folded chemokine on the basis of the published crystal structure (Protein Data Bank code 1080), thus confirming that the secondary structures of CTTC were intact (23, 45, 51).

### CTTC retains antimicrobial activity against the *B. anthracis* parent strain.

**FIG 3:**
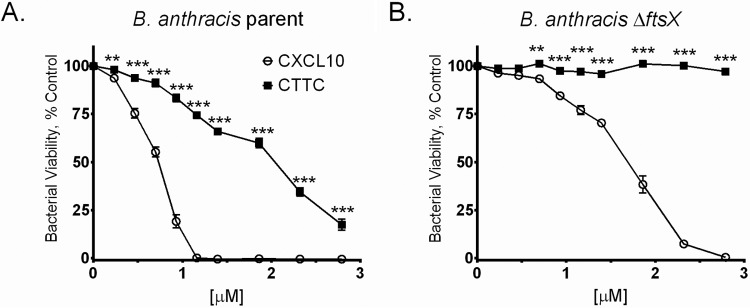
Antimicrobial activity of CTTC was retained, albeit less potently, against the *B. anthracis* parent strain but not against the *B. anthracis* Δ*ftsX* mutant. (A) CTTC killed the *B. anthracis* parent strain but was less potent than intact CXCL10 at concentrations ranging from 0 to 2.8 µM. (B) CTTC at 0 to 2.8 µM exhibited no antimicrobial effect against the *B. anthracis* Δ*ftsX* mutant, with a statistically significant difference observed between intact CXCL10 and CTTC. Bacterial viability was measured by alamarBlue reduction, and fluorescence is expressed as a percentage of that of the strain-specific untreated control. Data points represent mean values ± the standard errors of the means; *n* = 3 separate experiments using triplicate wells in each experiment. **, *P* ≤ 0.001; ***, *P* < 0.0001

Various concentrations of CXCL10 and CTTC were compared for antimicrobial activity against the *B. anthracis* parent strain. Micromolar concentrations were used instead of the previously published micrograms per milliliter because of the mass difference between CXCL10 and CTTC (35). Concentration-dependent killing of the *B. anthracis* parent strain was observed, with no viable bacteria detected at concentrations ≥1.2 µM CXCL10. CTTC also exhibited concentration-dependent killing but was less potent than intact CXCL10 (Fig. 3A). A statistically significant difference in antimicrobial activity between CXCL10 and CTTC was observed at all of concentrations tested. Thus, in the absence of the amphipathic α-helix, CTTC retained some antimicrobial activity, suggesting that the N terminus and other portions of CXCL10 could kill *B. anthracis* by a mechanism that was independent of the presence of the C-terminal α-helix.

### The *B. anthracis* Δ*ftsX* mutant is not susceptible to CTTC.

Based on previous studies in our laboratory (35), *B. anthracis* Δ*ftsX* was relatively resistant to CXCL10 when compared with the parent strain. However, this resistance could be overcome by higher concentrations of CXCL10 (Fig. 3B). In a previously published study, we showed that complementation of the *B. anthracis* Δ*ftsX* mutant with plasmid pUVA113, encoding an isopropyl-β-d-thiogalactopyranoside (IPTG)-inducible wild-type *ftsX* gene, restored susceptibility to CXCL10 compared to that of the Δ*ftsX* mutant complemented with the empty control vector pUTE973 (35). Therefore, to investigate whether the CXCL10 C-terminal α-helix plays a role in this killing, the antimicrobial activity of CTTC against the *B. anthracis* Δ*ftsX* mutant was assessed. In the absence of both FtsX and the CXCL10 α-helix, no antimicrobial activity was observed (Fig. 3B). A statistically significant difference between the activity of CXCL10 and that of CTTC against the Δ*ftsX* mutant was observed at all concentrations above 0.70 µM. The *ftsX*-complemented strain *B. anthracis* Δ*ftsX*/pUVA113 was susceptible to CTTC, whereas the Δ*ftsX* mutant complemented with empty vector plasmid pUTE973 remained resistant (see Fig. S1 in the supplemental material). These results suggested at least two possible mechanisms by which CXCL10 kills *B. anthracis*. One mechanism is mediated through the interaction between the N-terminal region of CXCL10 and bacterial FtsX and does not require the C-terminal α-helix (Fig. 3A); and the other, observed at higher concentrations of intact CXCL10 and in the absence of bacterial FtsX, requires the presence of the C-terminal α-helix (Fig. 3B).

### *B. anthracis* FtsE Walker motif mutant strain.

To test whether an active FtsE/X complex is necessary for CXCL10-mediated antimicrobial activity, an FtsE mutant with point mutations in the Walker A and B motifs was generated (52). FtsE/X is encoded within a single operon, and point mutations within the *ftsE* gene Walker motifs disrupt the function of FtsE without interfering with the expression of FtsX (40). Walker motifs are highly conserved between bacterial species and are involved in ATP binding and hydrolysis. Point mutations within Walker motifs are well documented to result in loss of the protein energy providing function (40, 42, 43, 49, 53, 54). Point mutations in *ftsE* resulted in two amino acid substitutions, K123A and D481N. The resulting mutant strain was designated *B. anthracis* *ftsE*(K123A/D481N). A genetically complemented *B. anthracis* *ftsE*(K123A/D481N) strain was generated. The wild-type *B. anthracis* parent strain *ftsE* gene was inserted into plasmid pUTE973 (55) to generate plasmid pUVA424, in which *ftsE* was under the control of an IPTG-inducible promoter. Because the *B. anthracis* *ftsE*(K123A/D481N) mutant was generated by chromosomal mutations, *B. anthracis* *ftsE*(K123A/D481N)/pUVA424 carried both mutated and wild-type copies of *ftsE.*

**FIG 4:**
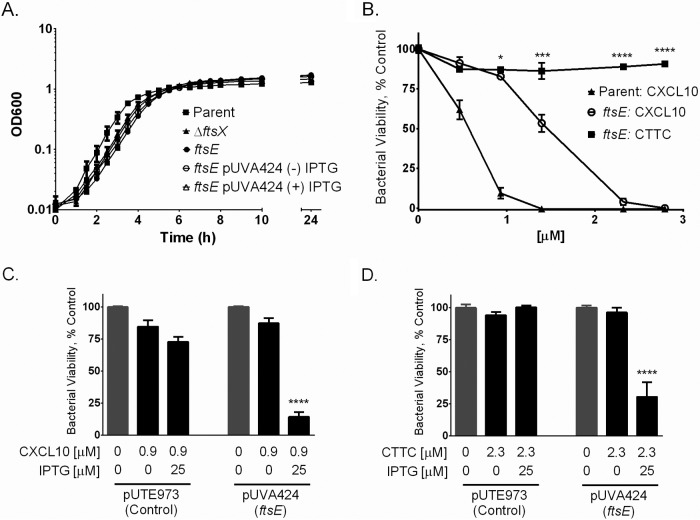
The *B. anthracis* *ftsE*(K123A/D481N) mutant was resistant to CXCL10 and CTTC, in contrast to the parent strain. (A) The *B. anthracis* *ftsE*(K123A/D481N) mutant (designated as "*ftsE*" in this figure) had a slightly longer lag phase prior to initiating log-phase growth than the *B. anthracis* parent strain, a growth characteristic similar to that observed in the *B. anthracis* Δ*ftsX* mutant (35). (B) The *B. anthracis* *ftsE*(K123A/D481N) mutant was relatively resistant to CXCL10 and resistant to CTTC at all of the concentrations tested, from 0 to 2.8 µM. Results were similar to those obtained with the *B. anthracis* Δ*ftsX* mutant. A statistically significant difference between CXCL10 and CTTC was observed at concentrations of ≥0.9 µM. (C) The *B. anthracis* *ftsE*(K123A/D481N) mutant transformed with IPTG-inducible plasmid pUTE973 (empty vector control) exhibited resistance to 0.9 µM CXCL10 in the absence or presence of 25 µM IPTG. Transformation of the *B. anthracis* *ftsE*(K123A/D481N) mutant with IPTG-inducible plasmid pUVA424 (*ftsE* complementation vector) resulted in restoration of susceptibility to 0.9 µM CXCL10 only upon treatment with 25 µM IPTG to induce gene expression. (D) The *B. anthracis* *ftsE*(K123A/D481N) mutant transformed with plasmid pUTE973 exhibited resistance to 2.3 µM CTTC in the absence or presence of 25 µM IPTG. The *B. anthracis* *ftsE*(K123A/D481N) mutant transformed with pUVA424 exhibited susceptibility to 2.3 µM CTTC only upon the addition of 25 µM control. Data points represent mean values ± the standard errors of the means; *n* = 3 separate experiments using IPTG to induce gene expression. Bacterial viability was measured by alamarBlue reduction, and fluorescence is expressed as a percentage of that of the strain-specific untreated triplicate wells in each experiment. *, *P* ≤ 0.01; ***, *P* ≤ 0.0001; ****, *P* < 0.0001.

Phenotypically, the *B. anthracis* parent strain grew as long, smooth chains of bacilli that were not affected by the presence and/or expression of pUTE973 or pUVA424 (see Fig. S2A to F in the supplemental material). In comparison, the *B. anthracis* *ftsE*(K123A/D481N) mutant bacilli resembled the *B. anthracis* Δ*ftsX* mutant (35), with a “kinked” appearance (see Fig. S2G). Addition of IPTG alone in the absence of any plasmids had no effect on the mutant phenotype of *B. anthracis* *ftsE*(K123A/D481N) (see Fig. S2J). The similarity in morphology between the *ftsE*(K123A/D481N) and Δ*ftsX* strains was as expected for mutations within the same functional complex. This morphological phenotype was also observed in similarly constructed *B. subtilis* mutants (40). The *B. anthracis* *ftsE*(K123A/D481N) mutant carrying the empty control vector (pUTE973) or an *ftsE* complementation plasmid (pUVA424) with no IPTG induction had the morphological phenotype of the *ftsE*(K123A/D481N) mutant strain (see Fig. S2H, I, and K). Induction of *ftsE* gene expression with IPTG generated a morphological phenotype similar to that of the *B. anthracis* parent strain (see Fig. S2L). A 24-h growth curve revealed that the *ftsE*(K123A/D481N) mutant strain had a slightly longer lag phase than the parent strain prior to initiating log-phase growth (Fig. 4A). The kinked morphology of *ftsE*(K123A/D481N) and that previously reported for Δ*ftsX* (35) suggested that FtsE and FtsX play a key role in cell wall synthesis and/or cell division, as has been observed in other bacterial species (39, 56, 57).

### The *B. anthracis ftsE*(K123A/D481N) mutant is resistant to CXCL10 and CTTC.

To test the potential role of FtsE, as part of an active FtsE/X complex, in CXCL10-mediated antimicrobial activity, the *B. anthracis* *ftsE*(K123A/D481N) mutant was exposed to CXCL10 or CTTC at various concentrations. The *B. anthracis ftsE*(K123A/D481N) mutant exhibited greater resistance to CXCL10-mediated killing compared to the parent strain (Fig. 4B). This resistance of the *ftsE*(K123A/D481N) mutant was overcome by higher concentrations of CXCL10 (Fig. 4B), similar to what was observed with the Δ*ftsX* mutant (Fig. 3B). The *B. anthracis* *ftsE*(K123A/D481N) mutant strain was resistant to CTTC at all of the concentrations tested (Fig. 4B), similar to the Δ*ftsX* mutant (Fig. 3B). IPTG-induced expression of the *ftsE*-containing vector (pUVA424) in the *ftsE*(K123A/D481N) mutant strain restored susceptibility to CXCL10 at 0.9 µM (Fig. 4C) and to CTTC at 2.3 µM (Fig. 4D). In contrast, IPTG induction of the empty vector control (pUTE973) in the *ftsE*(K123A/D481N) mutant strain did not restore susceptibility to CXCL10 or CTTC (Fig. 4C and D). These data suggest that a functional FtsE/X complex is important for the antimicrobial effect of CXCL10. Importantly, the *ftsX* gene was intact (i.e., wild type) in the *ftsE*(K123A/D481N) mutant strain in these experiments. Therefore, the data indicate that interaction of CXCL10 or CTTC with *B. anthracis* FtsX was not, in and of itself, sufficient for killing. Instead, an active FtsE/X complex was necessary for FtsX-mediated killing. Table 1 summarizes the calculated 50% effective concentration (EC_50_) values of CXCL10 and CTTC against the *B. anthracis* parent, Δ*ftsX*, and *ftsE*(K123A/D481N) strains.

**TABLE 1:** Calculated EC_50_ values of CXCL10 and CTTC for the *B. anthracis* parent, Δ*ftsX*, and *ftsE*(K123A/D481N) strains^a^

Strain	EC_50_ (µM) for:		Fold change
	CXCL10	CTTC	
Parent	0.68 ± 0.03	1.83 ± 0.07	2.7
Δ*ftsX* mutant	1.59 ± 0.05	NA^b^	NA
*ftsE*(K123A/D481N) mutant	1.39 ± 0.07	NA	NA

a The fold difference between the EC_50_ values of CXCL10 and CTTC for the *B. anthracis* parent strain is shown. The mutant strains showed no susceptibility to CTTC; thus, there is no EC_50_ or fold change compared to CXCL10.

b NA, not applicable because no killing was observed.

### *B. anthracis* parent strain membrane depolarization in response to CXCL10 or CTTC exposure.

The CXCL10 C-terminal α-helix does not appear to be required for the FtsE/X-mediated antimicrobial activity of CXCL10 in *B. anthracis*. However, it is likely that it plays a role in the non-FtsE/X-dependent mechanism of killing. Therefore, membrane depolarization studies were conducted to further investigate the role of the amphipathic α-helix of CXCL10. Initially, a peptide representing the CXCL10 α-helix (aa 54 to 77) was synthesized to test whether it has antimicrobial activity and depolarizes the membrane. CD studies revealed that this peptide formed an α-helix in the presence of trifluoroethylene, a membrane mimetic, at a concentration that was toxic to *B. anthracis* (data not shown). However, the C-terminal peptide did not form an α-helix in a buffer suitable for antimicrobial testing (data not shown). Therefore, a comparison of the effects of intact CXCL10 and CTTC on membrane depolarization was used to assess the effect of the α-helix (or lack thereof).

The *B. anthracis* parent strain was preloaded with the fluorescent dye 3,3′-dipropylthiadicarbocyanine (diSC3-5). The fluorescence of diSC3-5 is quenched upon cellular uptake by the negative membrane potential; membrane depolarization releases diSC3-5, which produces a fluorescent signal upon release. The fluorescent signal elicited by a test molecule is compared to that resulting from complete depolarization by the addition of the bee venom peptide melittin at 20 µM (58). The percentage of depolarization could then be calculated for each antimicrobial test molecule (59). Concurrently, the number of CFU per milliliter was determined to assess bacterial survival in this assay.

**FIG 5:**
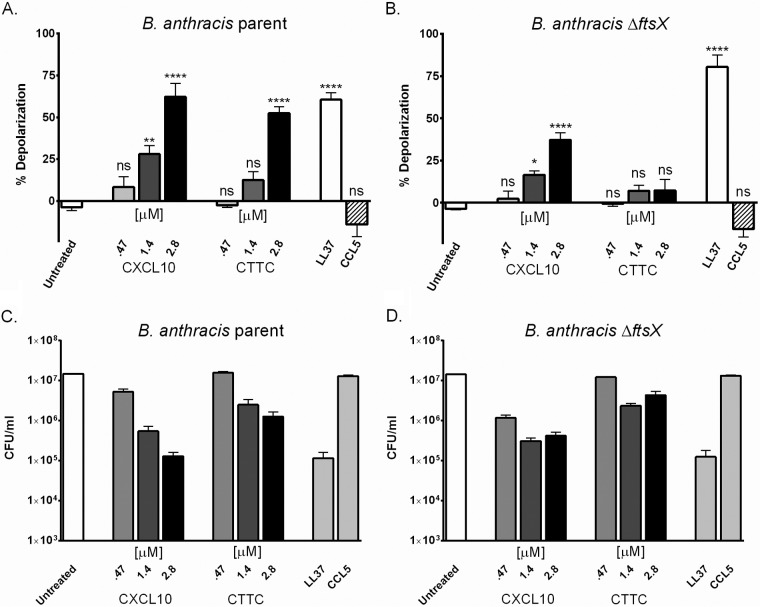
Membrane depolarization occurs after exposure of *B. anthracis* to CXCL10. (A) *B. anthracis* parent strain membrane depolarization occurs after exposure to CXCL10 or CTTC. (B) *B. anthracis* Δ*ftsX* mutant membrane depolarization occurs upon exposure to CXCL10 but not upon exposure to CTTC. The positive control (LL37 at 25 µM) induced membrane depolarization. Exposure to the negative control (CCL5 at 2.8 µM) did not cause membrane depolarization (*n* = 4 or 5 separate experiments). (C) Killing of the bacteria by CXCL10 or CTTC was correlated with the membrane depolarization assay results obtained under the same conditions (*n* = 4 or 5 separate experiments). Numbers of CFU per milliliter are plotted on a log scale. (D) Treatment of the *B. anthracis* Δ*ftsX* mutant with CXCL10 or CTTC at the concentrations indicated results in less killing than treatment of the parent strain shown in panel A (*n* = 4 or 5 separate experiments). Incubation with the positive control (LL37) resulted in less survival of both strains than of the untreated control strain. The negative control (CCL5) was not bactericidal (*n* = 4 or 5 separate experiments). Statistical analysis was performed by two-way ANOVA. ns, not significant; *, *P* ≤ 0.01; **, *P* ≤ 0.001; ***, *P* ≤ 0.0001; ****, *P* < 0.0001.

LL37, a cationic antimicrobial peptide that causes membrane disruption in bacteria, was used as a positive control at 25 µM (33). LL37 produced comparable degrees of membrane depolarization in both the parent and Δ*ftsX* mutant strains (Fig. 5A and B). CCL5, which has a molecular mass and charge similar to those of CXCL10 but exhibits no antimicrobial activity against *B. anthracis* (17), was used as a negative control. Exposure of the *B. anthracis* parent and Δ*ftsX* mutant strains to the same concentration of CCL5 as CXCL10 did not cause membrane depolarization (Fig. 5A and B).

The membrane of the *B. anthracis* parent strain underwent depolarization in the presence of CXCL10. The extent of depolarization increased with increasing concentrations of CXCL10 (Fig. 5A). Depolarization occurred to a lesser but statistically significant degree in the presence of CTTC (Fig. 5A). The degree of depolarization also increased with increasing concentrations of CTTC (Fig. 5A). Statistical analysis was conducted by comparing untreated and treated groups through a two-way analysis of variance (ANOVA). The effect of CTTC on the parent strain may indicate that some other portion of CXCL10, not just the α-helix, can produce depolarization or that cell lysis mediated through the FtsE/X-mediated pathway also leads to depolarization. The corresponding viability from CFU determination was plotted on a log scale and showed that the cells died upon exposure to either CXCL10 or CTTC (Fig. 5C). Because of an inoculum effect resulting from the higher bacterial concentration used in this particular assay relative to the concentration of CXCL10 or CTTC, the degree of bacterial killing was less than that observed in the standard antimicrobial assay shown in Fig. 3.

### Membrane depolarization of the *B. anthracis* Δ*ftsX* mutant occurs only in the presence of the CXCL10 α-helix.

CXCL10 caused significant membrane depolarization of the *B. anthracis* Δ*ftsX* mutant, in contrast to the untreated control (Fig. 5B). However, no significant membrane depolarization was observed when the *B. anthracis* Δ*ftsX* mutant was treated with CTTC (Fig. 5B). These data suggested that the membrane depolarization induced by CTTC in the parent strain was dependent upon the presence of FtsX. Thus, an FtsE/X-dependent pathway is likely to lead to a lack of cell integrity with concomitant membrane disruption. A decrease in bacterial viability was correlated with membrane depolarization upon incubation with CXCL10; a less pronounced decrease in viability was observed with CTTC (Fig. 5D). These results indicated that the C-terminal α-helix of CXCL10 was capable of causing membrane depolarization and that its activity was independent of FtsX.

## DISCUSSION

The antimicrobial activity of the interferon-inducible ELR(−) CXC chemokine CXCL10 has been demonstrated against a broad range of Gram-positive (including *B. anthracis*, *L. monocytogenes*, and *S. aureus*) and Gram-negative (*E. coli* and *Pseudomonas aeruginosa*) pathogens, but the exact mechanism(s) of action has remained unclear (14–16, 18, 19). In the present study, we showed that CXCL10 can kill *B. anthracis* through multiple mechanisms. One mechanism involves the bacterial FtsE/X complex and is mediated through interaction with the N-terminal region(s) of CXCL10. It is independent of the amphipathic C-terminal α-helix. Another mechanism is FtsE/X independent and is mediated by the depolarization of the cytoplasmic membrane by the CXCL10 C-terminal α-helix. Such findings are novel and unexpected in the chemokine field and open up a number of mechanistic possibilities for how chemokines target microbes beyond nonspecific membrane disruption, while also supporting defensin-like antibacterial targeting as a second mechanism.

**FIG 6:**
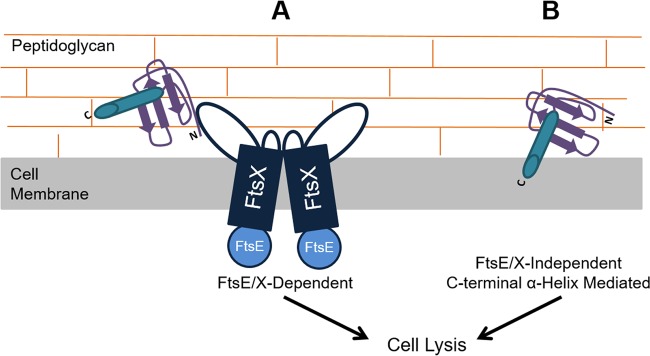
Hypothetical model of the bifunctional antimicrobial activity of CXCL10 against *B. anthracis*. (A) FtsE/X-mediated antimicrobial activity of CXCL10. CXCL10 elicits an antimicrobial effect against *B. anthracis* via an FtsE/X-dependent pathway through the N-terminal portion and/or other regions of CXCL10, independently of the C-terminal α-helix. Cell death results through lysis. (B) FtsE/X-independent antimicrobial activity of CXCL10. CXCL10 interacts with *B. anthracis* in an FtsX-independent manner to cause bacterial killing, likely through interaction or insertion of the C-terminal α-helix into the bacterial membrane, resulting in cell lysis. While the mechanistic pathways are different, both appear to lead to the same result of cell lysis.

Previous electron microscopy studies have shown that CXCL10 localizes to the bacterial membrane in vegetative cells and to the internal coat-cortex interface in spores (17). That an extracellular region of FtsX, a membrane protein, had limited similarity to CXCR3 suggested a possible interaction with CXCL10 through receptor mimicry. This observation led to the hypothesis that the N terminus of CXCL10 is important for interaction with the target microorganism through that region of FtsX, especially since it is the portion of the chemokine that facilitates interaction with CXCR3. A peptide competition assay showed the CXCR3-like region of FtsX was indeed important in interacting with CXCL10 and mediating antimicrobial activity. We then tested CTTC to determine if the N-terminal region of CXCL10 promotes antimicrobial activity or if the C-terminal α-helix of CXCL10 is necessary for killing. CTTC was less active against the *B. anthracis* parent strain and did not kill the Δ*ftsX* mutant strain. The EC_50_ values indicated that CTTC is 2.7-fold less effective than CXCL10 at killing the parent strain and lacks the ability to kill the Δ*ftsX* mutant strain altogether (Table 1). These results indicated that an FtsX-dependent mechanism of killing was independent of the CXCL10 C-terminal α-helix, highlighting a novel interaction between the CXCR3 receptor-binding region(s) of CXCL10 with bacterial FtsX (Fig. 6A), as chemokine antimicrobial activity has previously been hypothesized to be mediated through the C-terminal α-helix (19, 33, 34). The finding that other portions of CXCL10 may mediate killing represents a paradigm shift and opens avenues for the study and development of new therapies based on chemokine architecture.

FtsE/X is a unique bacterial complex that warrants further study as an antimicrobial target since its pivotal role in PG processing through hydrolase activation is widely conserved among bacterial species (38–41, 43, 60). Interestingly, there are differences between Gram-positive and Gram-negative bacteria in the roles of FtsE/X (37–41, 49). In Gram-positive bacteria, namely, *Bacillus* spp., there appear to be redundancies in the enzymatic activities involved in PG processing during cellular elongation, such that a disruption in one pathway could result in cell wall instability and bacterial cell lysis (44, 61, 62). Notably, the *B. anthracis* *ftsE*(K123A/D481N) mutant in our study exhibited a morphology similar to that previously reported for the Δ*ftsX* mutant, in which bacilli appeared “kinked,” with frequent curves and sharp angles among the growing chains, consistent with an alteration in cell wall processing (35, 39, 56, 57). The interaction of CXCL10 with FtsE/X may provoke or disrupt PG processing from which the bacteria cannot recover, resulting in bacterial cell death. Our results determined that CXCL10 FtsX-dependent antimicrobial activity requires both FtsX and a functional FtsE ATP-binding protein, thus implicating an energy requirement for the CXCL10-mediated mechanism. This report is the first description of an active FtsE/X complex contributing to the bactericidal effect of an antimicrobial molecule. The membrane disruption studies described here show that depolarization also occurred through the FtsX-mediated mechanism. Given the role of FtsE/X in PG processing, it is possible that CXCL10 provokes PG turnover, which leads to cell lysis through PG degradation and the loss of the cell’s ability to withstand environmental conditions. Studies are under way in our laboratory to investigate the nature of this interaction and how bacterial cell death may occur in response to CXCL10, potentially from provoking or disrupting the PG processing pathway. Understanding the function of the unique and widely conserved FtsE/X complex may lead to the utilization of this novel bacterial target for new antimicrobial therapies.

The FtsE/X-independent mechanism of killing by CXCL10 is likely mediated through the nonspecific interaction of its C-terminal α-helix with the bacterial membrane, similar to what has been described for cationic antimicrobial peptides such as LL37 or defensins (19, 33, 34) (Fig. 6B). The *B. anthracis* Δ*ftsX* mutant only undergoes membrane depolarization in the presence of intact CXCL10, suggesting that the amphipathic structural characteristics of the C-terminal region are responsible for membrane disruption and lysis. It appears that the CXCL10 C-terminal α-helix is not necessary for killing of the parent strain. However, in the absence of FtsX and at higher concentrations of CXCL10, death can occur through membrane disruption. When both mechanisms are present/active, CXCL10-mediated bacterial killing appears to be the most effective. Further study of the structural characteristics of CXCL10 and its related chemokines should lead to a better understanding of their biological activities, as well as a broader application of therapeutic potential for chemokine α-helices against a wide range of bacterial species.

Our findings represent an exciting advance in the understanding of how chemokines function as antimicrobial molecules, as well as in the study of host-pathogen interactions within the innate immune system. The primary significance is the new information involving the bifunctional activity of CXCL10, with mechanisms involving a previously unrecognized portion of CXCL10 that exhibits antimicrobial activity through interaction with the novel bacterial target FtsE/X and also the CXCL10 C-terminal α-helix-mediated antimicrobial killing of *B. anthracis*. Our research suggests that as chemokines are further studied as antimicrobial molecules, there will be heightened recognition of more specific mechanisms by which they kill microorganisms beyond nonspecific membrane targeting and disruption. Studies of the antimicrobial effects of chemokines are likely to reveal additional mechanisms of killing and lead to the development of novel small molecules as innovative therapies to treat a wide range of bacterial pathogens, including already established and emerging antibiotic-resistant organisms.

## MATERIALS AND METHODS

### Bacterial strains and culture conditions.

*B. anthracis* Sterne strain ATCC 7702 (pXO1^+^ pXO2^−^; American Type Culture Collection, Manassas, VA) was designated the parent strain and used in these experiments. The *B. anthracis* Δ*ftsX*, Δ*ftsX*/pUTE973, Δ*ftsX*/pUVA113, and *ftsE*(K123A/D481N) mutant strains were derived from the Sterne strain (35). Vegetative cells were prepared by inoculating *B. anthracis* parent strain spore stocks or *B. anthracis* Δ*ftsX* and *ftsE*(K123A/D481N) mutant vegetative cell frozen stocks into 10 ml of brain heart infusion (BHI) broth (Difco, Franklin Lakes, NJ) and incubating them overnight at 37°C with shaking (250 rpm). Mid-log-phase cultures were prepared on the next day by diluting the overnight cultures 1:20 in fresh BHI broth and incubating them at 37°C with shaking for ~2 h until an optical density at 600 nm between 0.6 and 0.65 was achieved, at which time the cultures were used for experiments. All laboratory work involving *B. anthracis* Sterne or Sterne-derived strains was approved by the University of Virginia Institutional Biosafety Committee. Biosafety level 2 practices were used for all work involving *B. anthracis* Sterne or Sterne-derived strains.

### Amino acid sequence alignment.

The National Library of Medicine Basic Local Alignment Search Tool (http://blast.ncbi.nlm.nih.gov/Blast.cgi) sequence alignment program was used to align Gram-positive and Gram-negative FtsX protein sequences with *B. anthracis* Sterne (parent) strain FtsX (see Text S1 in the supplemental material). The following species and FtsX identification codes were used to perform individual BLASTP analyses: *B. anthracis* Sterne (AAT57322.1), *B. subtilis* 168 (NP_391405.1), *M. tuberculosis* H37Rv (CAA49620.1), *S. pneumoniae* (AJD71681.1), *E. coli* F11 (EDV68789.1), and *K. pneumoniae* (KLA37751.1). Alignment of the full sequences of all six strains was conducted with the T-Coffee multiple-sequence alignment software (see Text S1) (http://tcoffee.crg.cat/apps/tcoffee/do:regular) and formatted with the Boxshade program (http://www.ch.embnet.org/software/BOX_form.html) (47, 48).

### Generation of *B. anthracis ftsE*(K123A/D481N) and plasmid vector pUVA424 for gene complementation studies with *B. anthracis ftsE*(K123A/D481N).

Point mutations of the *B. anthracis* gene *ftsE* were performed as previously described (35, 52). Briefly, *B. anthracis ftsE* was cloned into plasmid vector pGEM in α-select silver efficiency *E. coli* (Bioline, Taunton, MA) where Walker A and B mutations were performed (see Text S2A in the supplemental material). The mutated *ftsE* gene was amplified by PCR and sequenced to verify the mutations in the Walker A and B motifs. The bacterial strain with the two point mutations (K123A and D481N) in the *ftsE* Walker A and B motifs was designated *ftsE*(K123A/D481N).

Complementation of the *B. anthracis* *ftsE*(K123A/D481N) mutant strain was performed as previously described in detail (35). The generated *ftsE*(K123A/D481N) complementation plasmid (pUVA424) or an empty control plasmid vector (pUTE973) was electroporated into *B. anthracis* *ftsE*(K123A/D481N) bacilli. All isolates were tested and verified by PCR and sequencing.

### CXCL10 and CTTC.

Recombinant human CXCL10 was purchased from PeproTech (Rocky Hill, NJ). CXCL10 was dissolved in sterile H_2_O containing 0.3% (wt/vol) human serum albumin (HSA; Grifols, Los Angeles, CA) at a concentration of 1 mg ml^−1^ and stored at −80°C in 10-µl aliquots to limit freeze-thaw cycles to no more than one. The CTTC peptide was commercially synthesized and capped by amidation on the C terminus by United Biosystems (Herndon, VA) (see Text S2B in the supplemental material). Lyophilized CTTC was dissolved in sterile H_2_O at a concentration of 5 mg ml^−1^ and stored in 20-µl aliquots as described above. Sequence and structure verification was carried out as described in Text S2B.

### Antimicrobial assays and light microscopy studies.

Vegetative cells were diluted to approximately 2.5 × 10^5^ CFU ml^−1^ in fresh Dulbecco’s modified Eagle’s medium (Gibco-Invitrogen, Carlsbad, CA) containing 10% (vol/vol) fetal bovine serum (HyClone, Logan, UT) and the various concentrations of CXCL10 or CTTC to be tested. Aliquots of 100 µl were placed in triplicate wells of a 96-well plate and incubated at 37°C with 5% CO_2_ for approximately 4 h, unless otherwise noted, at which point alamarBlue dye (AbD Serotech, Oxford, United Kingdom) was added at a 1:10 dilution and allowed to reach visual saturation in an untreated control under protection from light. Active metabolism of the vegetative cells was quantified via the generation of a fluorescent signal from the reduction of the alamarBlue oxidation-reduction dye by measuring the fluorescence at 530-nm excitation and 590-nm emission wavelengths with a PerkinElmer Victor^3^ multilabel plate reader (PerkinElmer, Waltham, MA). The percentage of the control was calculated by comparison of the reading obtained with the chemokine-treated samples to that obtained with the corresponding untreated samples. Light microscopy was used for bacterial cell visualization. Camera control and image capture were performed with the QCapture pro-5.1 software as previously described (17, 35).

### Peptide competition assay.

The FtsX peptide and the control scrambled FtsX peptide were commercially synthesized by United Biosystems (Herndon, VA). The FtsX peptide (KVEQDVEIRVHIDPAAKEADQKKLEDD) was derived from the CXCR3-similar region noted in Fig. 1. The FtsX scrambled peptide (VEHRPQKADEDDALAKEKVKVIDQDIE) contained the same amino acids as the FtsX peptide but in random order. Each peptide was preincubated with 0.46 µM CXCL10 at a peptide-to-CXCL10 molar ratio of 10:1 or 20:1 for 30 min at 37°C. The peptide-CXCL10 mixtures were then used in a standard antimicrobial assay with equal volumes of buffer containing 0.3% HSA as a vehicle control as previously described (17). The antimicrobial assay incubation time was 2 h and was followed by the addition of alamarBlue dye. After visual saturation was reached, readings were obtained and the percentage of the control value was calculated.

### Membrane depolarization measurement.

After the exposure of vegetative cells to various micromolar concentrations of CXCL10 or CTTC, membrane depolarization was measured by a modification of a previously published protocol (59). Controls are discussed in Text S2C in the supplemental material. Briefly, *B. anthracis* cells were grown to mid-log phase as described above. Cells were collected and resuspended in respiration buffer, and 0.1 mM diSC3-5 (AnaSpec, Fremont, CA) was added to cell aliquots. Initial baseline fluorescence (*F*_0_) readings, test antimicrobial fluorescence (*F*) readings, and maximum depolarization (*F*_M_) readings were collected every 5 s for 150 to 300 s. Using the initial (*F*_0_), test (*F*), and final, maximum fluorescence readings (*F*_M_), the values of each group were averaged and the following calculation was used to determine percent depolarization by the test molecule compared to complete depolarization by melittin: % Depolarization = [(*F* − *F*_0_)/(*F*_M_ − *F*_0_)] × 100 (59). In parallel with the above-described depolarization experiments, the numbers of CFU per milliliter were determined to measure bacterial survival (see Text S2C).

### Statistical analyses.

Statistical analyses and graphing were performed with the GraphPad Prism 4.0 and 6.0 software. Experimental groups were analyzed with an unpaired, two-tailed Student *t* test or two-way ANOVA, as noted in the figure legends. Differences were considered significant at a *P* value of ≤0.05.

## SUPPLEMENTAL MATERIAL

Figure S1  The *B. anthracis* Δ*ftsX* mutant regains susceptibility to CTTC after genetic complementation with *ftsX*. The *B. anthracis* parent strain exhibited susceptibility to 2.3 µM CTTC with or without 50 µM IPTG present, while the *B. anthracis* Δ*ftsX* mutant was fully resistant to 2.3 µM CTTC. Testing of the *B. anthracis* Δ*ftsX* mutant carrying empty control vector pUTE973 resulted in retention of resistance to CTTC. Testing of the *B. anthracis* Δ*ftsX* mutant carrying *ftsX* complementation plasmid pUVA113 resulted in restoration of susceptibility to CTTC in the IPTG-induced sample but not the noninduced control. Download Figure S1, TIF file, 0.1 MB

Figure S2  The *B. anthracis* *ftsE*(K123A/D481N) mutant exhibits a “kinked” phenotype when observed by light microscopy. (A) The *B. anthracis* parent strain exhibited long, smooth chains of bacilli. (B, C) *B. anthracis* transformed with empty control vector pUTE973 retained the parent phenotype without and with induction by 50 µM IPTG. (D) Addition of 50 µM IPTG alone in the absence of all plasmids had no effect on parent strain morphology. (E, F) The presence and induction of *ftsE* complementation vector pUVA424 in the parent strain also elicited no morphological change. (G) *B. anthracis* *ftsE*(K123A/D481N) exhibited a “kinked” phenotype similar to that previously observed in the *B. anthracis* Δ*ftsX* mutant (35). (H, I) *B. anthracis* *ftsE*(K123A/D481N) transformed with empty control vector pUTE973 retained the “kinked” characteristics of the mutant strain with or without induction by 50 µM IPTG. (J) Addition of IPTG alone in the absence of any plasmids had no effect on the mutant phenotype of *B. anthracis* *ftsE*(K123A/D481N). (K) *B. anthracis* *ftsE*(K123A/D481N) transformed with *ftsE* complementation vector pUVA424 exhibited a “kinked” appearance in the absence of IPTG. (L) *B. anthracis* *ftsE*(K123A/D481N) transformed with pUVA424 exhibited a phenotype similar to that of the *B. anthracis* parent strain only when *ftsE* gene expression was induced by 50 µM IPTG. Representative fields from three independent experiments are shown at ×200 magnification. Download Figure S2, TIF file, 1.4 MB

Text S1  Supplemental bacterial FtsX amino acid sequence alignments. An amino acid sequence alignment using the *B. anthracis* Sterne strain FtsX as the reference protein was conducted with selected Gram-positive, acid-fast, and Gram-negative bacterial species. FtsE/X is widely conserved among bacterial species, with similarity levels ranging between 46 and 75%, as determined by BLASTP analysis and amino acid sequence alignment. The species (FtsX identification codes) used to perform individual BLASTP analyses were *B. anthracis* Sterne (AAT57322.1), *B. subtilis* 168 (NP_391405.1), *M. tuberculosis* H37Rv (CAA49620.1), *S. pneumoniae* (AJD71681.1), *E. coli* F11 (EDV68789.1), and *K. pneumoniae* (KLA37751.1). Full-sequence alignment of all six strains was conducted with the T-Coffee multiple-sequence alignment software (http://tcoffee.crg.cat/apps/tcoffee/do:regular) and formatted with the Boxshade program (http://www.ch.embnet.org/software/BOX_form.html) (47, 48). Download Text S1, DOCX file, 0.02 MB

Text S2  Additional details of the materials and methods used in these studies. (A) Generation of the *B. anthracis* *ftsE*(K123A/D481N) mutant strain and plasmid vector pUVA424 for gene complementation studies with the *B. anthracis* *ftsE*(K123A/D481N) mutant. (B) CTTC verification by CD studies. (C) Membrane depolarization measurement studies. Download Text S2, DOCX file, 0.02 MB
