# Supplementary figures and images for: CXCL10 Acts as a Bifunctional Antimicrobial Molecule against Bacillus anthracis
Source: mBio. 2016 May 10;7(3):e00334-16. doi: 10.1128/mBio.00334-16 (PMC4959661; doi:10.1128/mBio.00334-16)

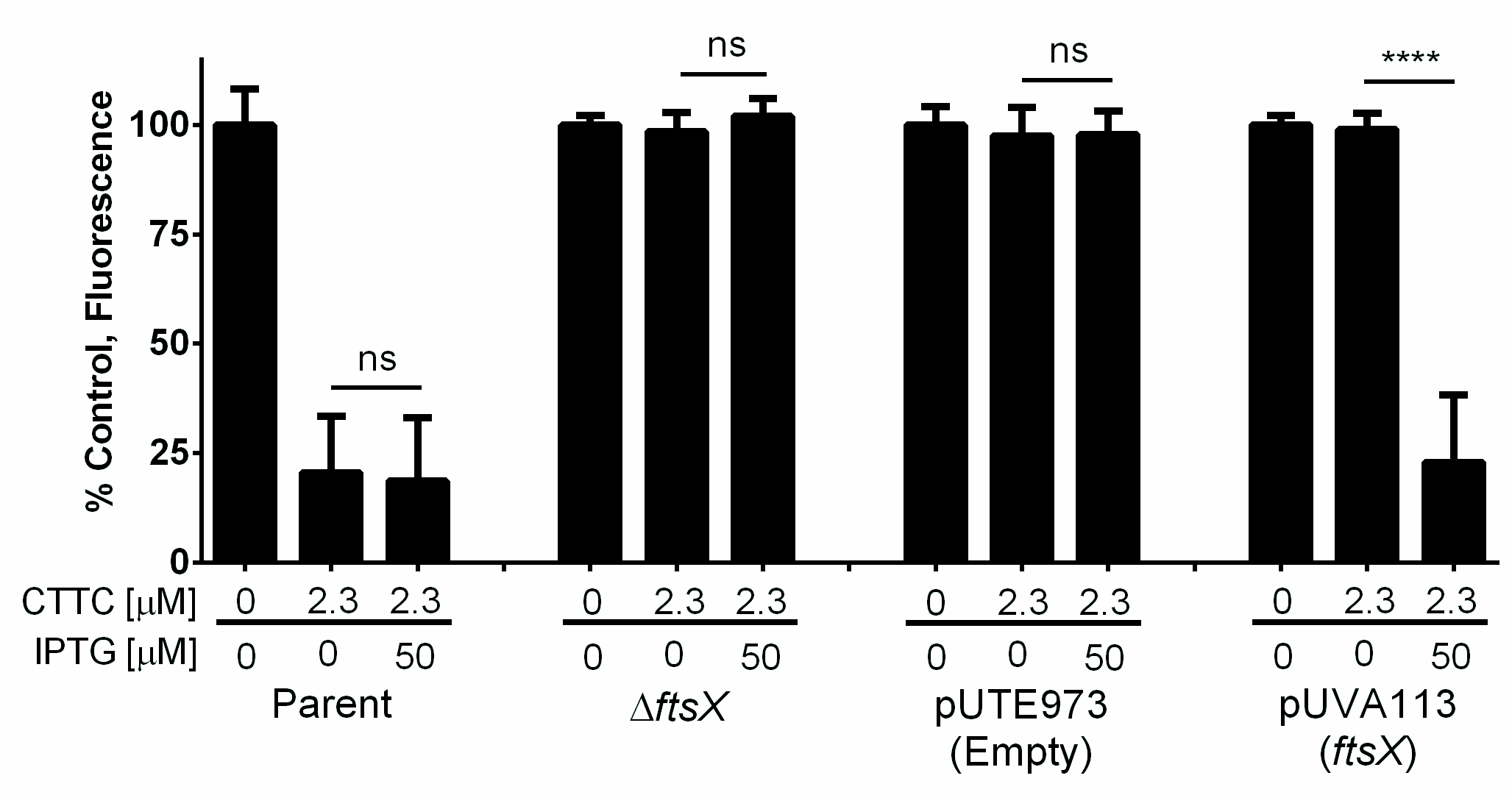

Supplement: Figure S1 — The B. anthracis ΔftsX mutant regains susceptibility to CTTC after genetic complementation with ftsX. The B. anthracis parent strain exhibited susceptibility to 2.3 µM CTTC with or without 50 µM IPTG present, while the B. anthracis ΔftsX mutant was fully resistant to 2.3 µM CTTC. Testing of the B. anthracis ΔftsX mutant carrying empty control vector pUTE973 resulted in retention of resistance to CTTC. Testing of the B. anthracis ΔftsX mutant carrying ftsX complementation plasmid pUVA113 resulted in restoration of susceptibility to CTTC in the IPTG-induced sample but not the noninduced control. Download [file mbo002162807sf1.tif]

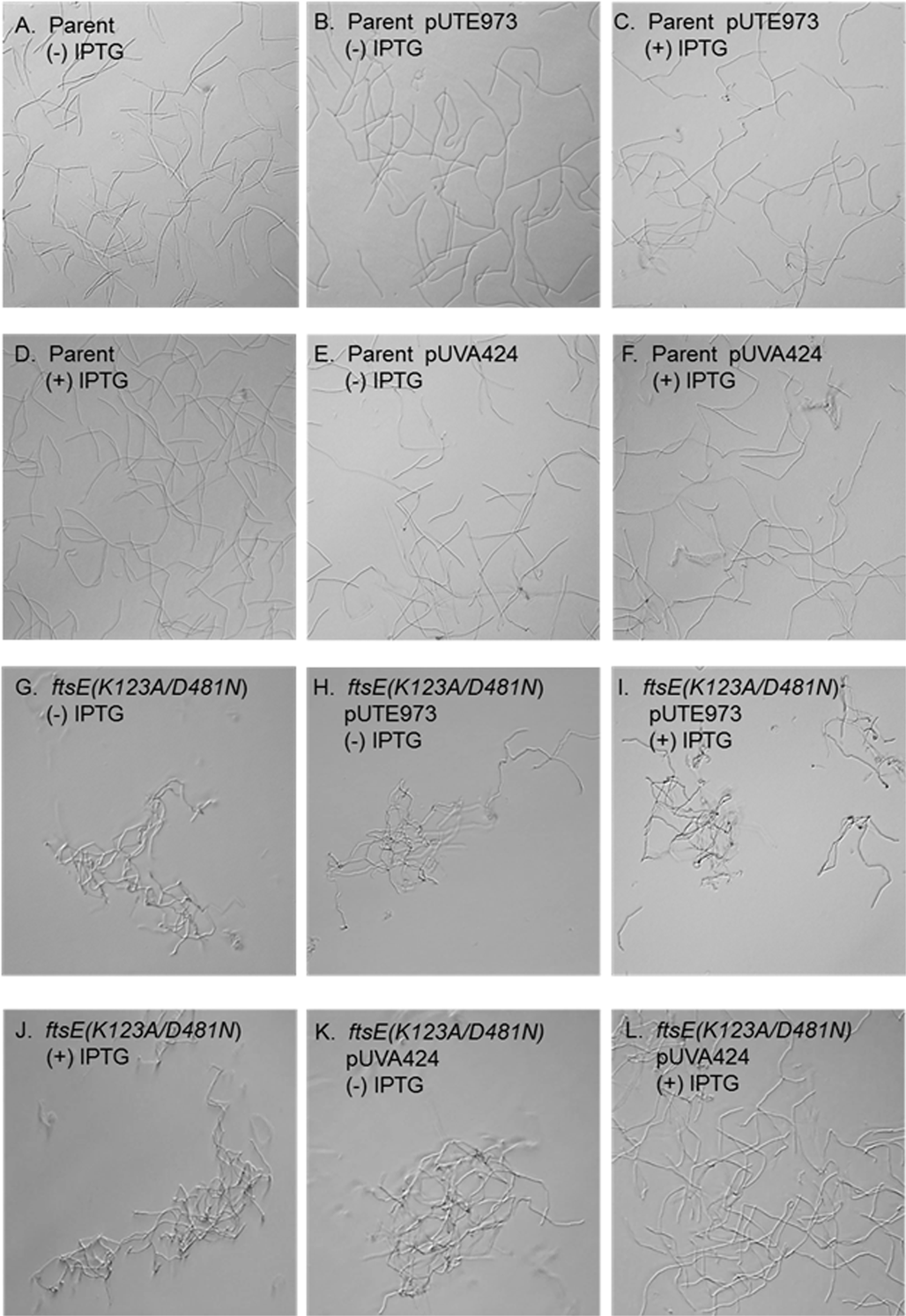

Supplement: Figure S2 — The B. anthracis ftsE(K123A/D481N) mutant exhibits a “kinked” phenotype when observed by light microscopy. (A) The B. anthracis parent strain exhibited long, smooth chains of bacilli. (B, C) B. anthracis transformed with empty control vector pUTE973 retained the parent phenotype without and with induction by 50 µM IPTG. (D) Addition of 50 µM IPTG alone in the absence of all plasmids had no effect on parent strain morphology. (E, F) The presence and induction of ftsE complementation vector pUVA424 in the parent strain also elicited no morphological change. (G) B. anthracis ftsE(K123A/D481N) exhibited a “kinked” phenotype similar to that previously observed in the B. anthracis ΔftsX mutant (35). (H, I) B. anthracis ftsE(K123A/D481N) transformed with empty control vector pUTE973 retained the “kinked” characteristics of the mutant strain with or without induction by 50 µM IPTG. (J) Addition of IPTG alone in the absence of any plasmids had no effect on the mutant phenotype of B. anthracis ftsE(K123A/D481N). (K) B. anthracis ftsE(K123A/D481N) transformed with ftsE complementation vector pUVA424 exhibited a “kinked” appearance in the absence of IPTG. (L) B. anthracis ftsE(K123A/D481N) transformed with pUVA424 exhibited a phenotype similar to that of the B. anthracis parent strain only when ftsE gene expression was induced by 50 µM IPTG. Representative fields from three independent experiments are shown at ×200 magnification. Download [file mbo002162807sf2.tif]
