# Supplementary material for: CXCL10 Acts as a Bifunctional Antimicrobial Molecule against Bacillus anthracis
Source: mBio. 2016 May 10;7(3):e00334-16. doi: 10.1128/mBio.00334-16 (PMC4959661; doi:10.1128/mBio.00334-16)
Supplement: Text S1 — Supplemental bacterial FtsX amino acid sequence alignments. An amino acid sequence alignment using the B. anthracis Sterne strain FtsX as the reference protein was conducted with selected Gram-positive, acid-fast, and Gram-negative bacterial species. FtsE/X is widely conserved among bacterial species, with similarity levels ranging between 46 and 75%, as determined by BLASTP analysis and amino acid sequence alignment. The species (FtsX identification codes) used to perform individual BLASTP analyses were B. anthracis Sterne (AAT57322.1), B. subtilis 168 (NP_391405.1), M. tuberculosis H37Rv (CAA49620.1), S. pneumoniae (AJD71681.1), E. coli F11 (EDV68789.1), and K. pneumoniae (KLA37751.1). Full-sequence alignment of all six strains was conducted with the T-Coffee multiple-sequence alignment software (http://tcoffee.crg.cat/apps/tcoffee/do:regular) and formatted with the Boxshade program (http://www.ch.embnet.org/software/BOX_form.html) (47, 48). Download [file mbo002162807s1.docx]

**Text S1: Supplemental Bacterial FtsX Amino Acid Sequence Alignments**

Sequence alignment of FtsX from representative bacterial species

Ba: *B. anthracis* parent (Gram-positive)

Bs: *B. subtilis* (Gram-positive) 60% identity; 75% positive

Mt: *M. tuberculosis* (Acid-fast) 22% identity; 46% positive

Sp: *S. pneumoniae* (Gram-positive) 41% identity; 60% positive

Ec:  *E. coli* (Gram-negative) 28% identity; 49% positive

Kp: *K. pneumoniae* (Gram-negative) 26% identity; 47% positive

% Identity and % Positive are notated when compared to *B. anthracis* FtsX after Blastp analysis.

Red underline designates CXCR3-similar regions of *B. anthracis* parent strain FtsX.

Black highlighting designates conserved residues, gray highlighting designates characteristically similar residues.

Ba 1 MKAKTLSRHLR-------------------------------------------------
Bs 1 MIK-ILGRHLR-------------------------------------------------
Mt 1 MRFGFL---LN-------------------------------------------------
Sp 1 MIS-RFFRHLF-------------------------------------------------
Ec 1 MNKRDAINHIRQFGGRLDRFRKSVGGSGDGGRNAPKRAKSSPKPVNRKTNVFNEQVRYAF
Kp 1 MNKRDAMNQIRQFGSKFDRLRNAAGG-GGGGRNAPKRPKAAPNPASRKSNVFNEQVRYAW


Ba 12 -EGVKNLSRNGWMTFASVSAVTVTLLLVGVFLTAIMNMNHFATKVEQDVEIRVHIDPA--
Bs 11 -ESFKSLGRNTWMTFASISAVTVTLILVGVFLVIMLNLNNMATNAEKQVEIKVLIDLT--
Mt 9 -EVLTGFRRNVTMTIAMILTTAISVGLFGGGMLVVRLADSSRAIYLDRVESQVFLTEDVS
Sp 11 -EALKSLKRNGWMTVAAVSSVMITLTLVAIFASVIFNTAKLATDIENNVRVVVYIRKD--
Ec 61 HGALQDLKSKPFATFLTVMVIAISLTLPSVCYMVYKNVNQAATQYYPSPQITVYLQKT--
Kp 60 HGALQDLKSTPLATFLTVMVIAISLTLPSVCYMVYKNVSSAASQYYPSPQITVYLEKT--


Ba 69 -----AKEAD--------------QKKLEDDMSKIAKVESIKYSSKEEELKRLIKSLGDS
Bs 68 -----ADQKA--------------QDKLQNDIKELKGIQSVTFSSKEKELDQLVDSFGDS
Mt 68 ANDSSCDTTA--------------CKALREKIETRSDVKAVRFLNRQQAYDDAIRKFPQF
Sp 68 -----VEDNSQTIEKEGQTVTNNDYHKVYDSLKNMSTVKSVTFSSKEEQYEKLTEIMGDN
Ec 119 -----LDDDA--------------AAGVVAQLQAEQGVEKVNYLSREDALGEFRNWSG-F
Kp 118 -----LDDDA--------------AARVVGQLQAEQGVDKVNYLSRDEALGEFRNWSG-F


Ba 110 GKTFELFEQDNPLKNVFVVKAKE-P---TDTATIAKKIEKMQFVSNVQYGKGQVERLFDT
Bs 109 GKSLTMKDQENPLNDAFVVKTTD-P---HDTPNVAKKIEKMDHVYKVTYGKEEVSRLFKV
Mt 114 K---DV-AGKDSFPASFIVKLEN-P---EQHKDFDTAMKGQPGVLDVLNQKELIDRLFAV
Sp 123 WKIFE--GDANPLYDAYIVEANT-P---NDVKTIAEEAKKIEGVSEVQDGGANTERLFKL
Ec 159 GGALDM-LEENPLPAVAVVIPKLDFQGTESLNTLRDRITQINGIDEVRMDDSWFARLAAL
Kp 158 GGALDM-LEENPLPAVAIVVPKLDFQSTEALNTLRDRVSRIQGVDEVRMDDSWFARLSSL


Ba 166 VKTGRNIGIVLIAGLLFTAM-FLISNTIKITIYARSTEIEIMKLVGATNWFIRWPFLLEG
Bs 165 VGVSRNIGIALIIGLVFTAM-FLISNTIKITIFARRKEIEIMKLVGATNWFIRWPFFLEG
Mt 166 LDGLSNAAFAVALVQAIGAI-LLIANMVQVAAYTRRTEIGIMRLVGASRWYTQLPFLVEA
Sp 177 ASFIRVWGLGIAALLIFIAV-FLISNTIRITIISRSREIQIMRLVGAKNSYIRGPFLLEG
Ec 218 TGLVGRVSAMIG-VLMVAAVFLVIGNSVRLSIFARRDSINVQKLIGATDGFILRPFLYGG
Kp 217 TGLVGRVSAMIG-VLMVAAVFLVIGNSVRLSIFARRDTINVQKLIGATDGFILRPFLYGG


Ba 225 LFLGVLGSIIPI-GLILVTYNSLQGMFNEKLGGTIFELLPYSPFVFQLAGLLVLIGALIG
Bs 224 LLLGVFGSVIPI-ALVLSTYQYVIGWVVPKVQGSFVSLLPYNPFVFQVSLVLIAIGAVIG
Mt 225 MLAATMGVGIAVAGLMVVRALFLENALNQFYQANLIAKVDYADILFITPWLLLLGVAMSG
Sp 236 AFIGLLGAIAPS-VLVFIVYQIVYQSVNKSLVGQNLSMISPDLFSPLMIALLFVIGVFIG
Ec 277 ALLGFSGALLSL-ILSEILVLRLSSAVAE-VAQVFGTKFDINGLSFDECLLLLLVCSMIG
Kp 276 AMLGFSGAFLSL-ILSEILVMRLSSAVTE-VAKVFGTQFELSGLGFDECLLMLIVCSMIG


Ba 284 MWGSVMSIRR----FLKV
Bs 283 VWGSLTSIRK----FLRV
Mt 285 -LTAYLTLRL----YVRR
Sp 295 SLGSGISMRR----FLKI
Ec 335 WVAAWLATVQHLRHFTPE
Kp 334 WVAAWLATVQHLRHFTPD
