# Supplementary material for: CXCL10 Acts as a Bifunctional Antimicrobial Molecule against Bacillus anthracis
Source: mBio. 2016 May 10;7(3):e00334-16. doi: 10.1128/mBio.00334-16 (PMC4959661; doi:10.1128/mBio.00334-16)
Supplement: Text S2 — Additional details of the materials and methods used in these studies. (A) Generation of the B. anthracis ftsE(K123A/D481N) mutant strain and plasmid vector pUVA424 for gene complementation studies with the B. anthracis ftsE(K123A/D481N) mutant. (B) CTTC verification by CD studies. (C) Membrane depolarization measurement studies. Download [file mbo002162807s2.docx]

**Text S2: Supplemental Materials and Methods**

**Contents of Text S2**

1. Generation of *B. anthracis ftsE(K123A/D481N)* strain and the plasmid vector, pUVA424, for gene complementation studies in *B. anthracis ftsE(K123A/D481N)*
2. C-terminal truncated CXCL10 (CTTC) Verification
3. Membrane Depolarization Measurement
4. **Generation of *B. anthracis ftsE(K123A/D481N)* strain and the plasmid vector, pUVA424, for gene complementation studies in *B. anthracis ftsE(K123A/D481N)***

*B. anthracis ftsE* was cloned into the plasmid vector pGEM in α-select silver efficiency *E. coli* (Bioline, Taunton, MA) in preparation for PCR mediated point mutations the Walker A and Walker B motifs. The Walker A mutation (K123A) was performed using the forward primer: AGTGGAGCCGGGGCATCTACATTTATT and reverse primer: CGGTCCAACTACGTATACAAACTCACC. The Walker B mutation (D481N) was performed sequentially using the forward primer: GTCGTAATTGCCAATGAGCCAACA and reverse primer: TTTCGGCTTGTTTACAATTGCTCTC. The Walker motif mutated *ftsE* construct was then cloned into plasmid pRP1028 and placed in *E. coli* 138 in preparation for chromosomal integration through homologous recombination. *E. coli* 138 containing the pRP1028 *ftsE* plasmid was conjugated with *B. anthracis* parent strain to integrate the mutated *ftsE* into the *B. anthracis* chromosome. Plasmid pBKJ236 was introduced through electroporation to facilitate chromosomal recombination to obtain colonies with a single copy of the mutated *ftsE*. Individual colonies were isolated and screened for loss of erythromycin resistance. The *ftsE gene* was amplified by PCR and sequenced to verify the mutations in the Walker A and B motifs. The plasmid pBJK233 was lost spontaneously by multiple passages in media lacking tetracycline. This bacterial strain with the two point mutations (K123A and D481N) in the *ftsE* Walker A and Walker B motifs was designated *ftsE(K123A/D481N)*.

Gene complementation of *ftsE* in the *B. anthracis ftsE(K123A/D481N)* was performed as described in detail (34). Briefly, the native *B. anthracis* parent strain *ftsE* gene was amplified using a forward primer carrying a ribosome binding site and SphI restriction site (CCGGATACTCGAGTGAGGAGGAGGCTACCTTCAACAAGAGTGG) and a reverse primer carrying PspXI restriction site (GCCTAGGCATGCTTCACACCTTCTCGCAAATG). The *ftsE* PCR product was double digested with SphI-HF and PspXI, purified, and ligated into the empty plasmid vector pUTE973 that had been digested with SphI-HF and Salk-HF. The resulting vector was transformed into α-select silver efficiency *E. coli* and verified by sequencing. The verified plasmid was isolated and electroporated into GM119 *E. coli* to remove all methylation. The resulting *ftsE(K123A/D481N)* complementation plasmid (pUVA424), or the empty vector control plasmid (pUTE973), was then electroporated into the *B. anthracis ftsE(K123A/D481N)* bacterial strain. All isolates were tested and verified by PCR and sequencing.

1. **C-terminal truncated CXCL10 (CTTC) Verification**

The CTTC peptide, -VPLSRTVRCTCISISNQPVNPRSLEKLEIIPASQFCPRVEIIATMKKKGEKRCL-NH2, was commercially synthesized and capped by amidation on the C-terminus by United Biosystems (Herndon, VA). Sequence was confirmed by mass spectrometry conducted by United Biosystems. Lyophilized CTTC was dissolved in sterile H_2_O at a concentration of 5 mg ml^-1^ and stored in 20 μl aliquots as described above. Circular dichroism studies were conducted on 50-100 μM samples of CXCL10 and CTTC resuspended in sterile H_2_O to analyze secondary structure protein characteristics (AVIV 410 Circular Dichroism Spectropolarimeter, Lakewood, New Jersey). CD analysis of CTTC established that an α-helical structure was absent, as compared to the intact CXCL10 spectrum. Analyses were conducted using CDNN software (http://gerald-boehm.de/download/cdnn) and data were compared to the expected secondary structure prediction of CXCL10 and CTTC (https://www.predictprotein.org/) (PDB: 1080) (22, 44, 46). CD analysis of CTTC revealed an expected content of β-strands (39%), indicating that CTTC was properly folded.

1. **Membrane Depolarization Measurements**

Membrane depolarization was measured after exposure of vegetative cells to various μM concentrations of CXCL10 or CTTC using a modification of a published protocol (55). Controls included LL-37 as a positive control (15) and CCL5 as a negative control (17). LL-37 (Alpha Diagnostic International, San Antonio, Texas) was reconstituted in 0.1% BSA with 0.01% acetic acid and used at a final concentration of 25 μM. CCL5 (Peprotech, Rocky Hill, New Jersey) was reconstituted in 0.3% HSA and used at a final concentration of 2.8 μM.

Briefly, *B. anthracis* cells were grown to mid-log phase, as described above. Aliquots (1.5 ml) were centrifuged at 15,000 rpm, and the supernatant was discarded. Vegetative cells were resuspended in respiration buffer (5 mM HEPES, 20 mM glucose, pH= 7.4) to an optical density of 0.100 at 600 nm. A 1 μl aliquot of 0.1 mM 3,3’-dipropylthiadicarbocyanine (diSC3-5) (AnaSpec, Fremont, CA) was added to 1.5 ml aliquots of the resuspended cells, gently mixed, and then incubated in the dark at room temperature for 1 h. After incubation, a 100 μl aliquot of dye-loaded bacteria was placed into a 96-well black bottom plate (VWR, Radnor, PA). An initial baseline fluorescence reading (F_0_) of the sample well was measured every 5 seconds for a total of 120 seconds at 540-nm excitation and 680-nm emission using the Perkin-Elmer Victor^3^ multi-label plate reader. After collecting these baseline readings, the test antimicrobial molecule was added at various concentrations to the sample, and fluorescence (F) readings were collected every 5 seconds for 300 seconds. At this final time point, the bee venom peptide, melittin (Enzo Life Sciences, Farmingdale, NY), was added to a final concentration of 20 μM to complete depolarization and end point readings (maximum depolarization) (F_M_) were collected every 5 seconds for 300 seconds. Using the initial (F_0_), test (F), and final, maximum fluorescence readings (F_M_), the values of each group were averaged together, and the following calculation was used to determine percent depolarization by the test molecule compared to complete depolarization by melittin: % Depolarization = [(F-F_0_)/(F_M_-F_0_)]X100 (55).

In parallel with the above depolarization experiments, aliquots of samples prepared in the same way were collected (in the absence of melittin addition), and then diluted and inoculated onto BHI agar plates followed by for overnight incubation at 37°C to determine colony forming units ml^-1^ (CFU ml^-1^). All treated bacteria were accompanied by an untreated control for comparison of CFU ml^-1^.
